# Unlocking the Potential of Water-Insoluble Natural Polymers: Isolation, Characterization, and 2D NMR Quantification of *cis*-1,4-Poly-β-myrcene in Chios Mastic Gum

**DOI:** 10.1021/acs.jnatprod.5c00256

**Published:** 2025-04-14

**Authors:** Stavros Beteinakis, Eleni V. Mikropoulou, Dimitris Michailidis, Apostolis Angelis, Martina Haack, Marion Ringel, Thomas Brück, Dieter W. Brück, Jean-Hugues Renault, Alexios-Leandros Skaltsounis, Pedro Lameiras, Maria Halabalaki

**Affiliations:** † Division of Pharmacognosy and Natural Products Chemistry, Department of Pharmacy, 68993 National and Kapodistrian University of Athens, Panepistimioupoli Zografou, 15771 Athens, Greece; ‡ PharmaGnose S.A., 57th km Athens-Lamia National Road, 32011 Oinofyta, Greece; § Werner Siemens—Chair of Synthetic Biotechnology, Department of Chemistry, School of Natural Sciences, 9184 Technical University of Munich, 85748 Garching, Germany; ∥ 27078 University of Reims Champagne-Ardenne, ICMR 7312, 51687 Reims, France

## Abstract

Natural polymers have garnered attention due to their unique properties, i.e., structural versatility, biocompatibility, and modifiability. Recent efforts focus on sustainable raw materials to develop environmentally friendly processes and products that align with global sustainability goals. Among these, Chios mastic gum, derived from the mastic tree (*Pistacia lentiscus* var. *Chia*), is notable for its diverse food, pharmaceutical, and cosmetics applications. One of its key components is *cis*-1,4-poly-β-myrcene, a natural polyterpene polymer, constituting 20–30% of the resin’s composition. Despite its potential, the complex composition of Chios mastic gum poses challenges in extracting, isolating, and quantifying its polymeric content. NMR spectroscopy offers a nondestructive approach and may be instrumental in developing standardized methods for quantifying *cis*-1,4-poly-β-myrcene in Chios mastic gum. Such methods are vital for understanding the resin’s composition and exploring potential applications, particularly in sustainable materials and biomedical fields. This study addresses these challenges by producing a *cis*-1,4-poly-β-myrcene sample as a standard in quantification procedures. Centrifugal partition chromatography, a support-free liquid–liquid chromatography technique, was employed to purify the polymeric fraction. The polymer was then characterized through size exclusion chromatography and NMR methods, including DOSY and quantitative HSQC experiments, to facilitate an accurate analysis and open the door to further applications of this natural polymer.

## Graphical abstract

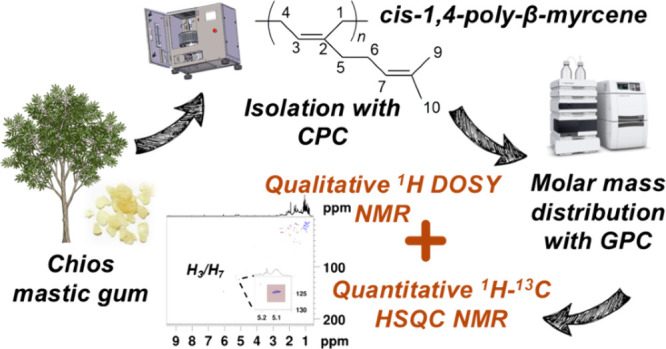

Natural polymers are large, chain-like molecules composed of repeating structural units, often derived from biological sources. Common examples of natural polymers include cellulose, proteins, and starch. However, in recent years, increasing attention has been paid to water-insoluble natural polymers such as polyisoprenes and polyterpenes due to their unique properties, including structural versatility, biocompatibility, easy accessibility, and modification.^1^ Over the last two decades, the use of sustainable raw materials for the development of environmentally friendly processes and products has been a major focus of both industry and academia, and it has been extensively promoted by national and international policymakers, such as the United Nations 2030 Agenda for Sustainable Development.^2^

Chios mastic gum (CMG), a resin obtained from the mastic tree (*Pistacia lentiscus* var. *Chia*), is well-known for its unique composition and wide range of uses in food, pharmaceuticals, and cosmetics.^3^ CMG forms a complex structure, with one of its key components, *cis*-1,4-poly-β-myrcene—a natural polyterpene polymer—comprised of approximately 20–30% of the total resin’s weight.^4^ CMG is also rich in terpenes and particularly triterpenic acids, with the isomers masticadienonic and isomasticadienonic acids, mastic’s most characteristic compounds, representing close to 30% of the crude resin.^5^ However, the complex composition of mastic gum introduces several issues in sample handling, extraction, isolation, and quantification of its components. This is particularly true for the resin’s natural polymer, whose water-insoluble nature poses a real challenge for further study and exploitation.^3^

Among the first efforts to attempt the removal of mastic polymer was that described by Barton and Seoane,^6^ who reported the decantation of the “insoluble residue” with a mixture of Et_2_O and MeOH. Nevertheless, the first study to examine the structure of mastic polymer was that by van der Berg et al.,^7^ where the authors used a similar precipitation procedure, employing CH_2_Cl_2_ and MeOH to isolate the polymeric fraction. Afterward, size exclusion chromatography (SEC), spectroscopic, and spectrometric methods were utilized to investigate the polymer’s molar mass and monomeric units; finally, through this effort, the polymer’s structure was identified as *cis*-1,4-poly-β-myrcene. More recently, Sharifi et al.,^8^ in an attempt to assess the bioactivity of polymers found in different *Pistacia* sp., isolated the polymer of *P. lentiscus* with an identical precipitation workflow. In this work, among others, the polymer content in mastic gum was determined solely on the basis of mass, while gel permeation chromatography (GPC), a type of SEC commonly employed in polymer analysis, was used for characterizing the molar mass distribution.

Thereafter, many research groups working with CMG in the natural products sector have employed similar procedures to “discard” the insoluble and somewhat inconvenient polymeric fraction, always with the use of high quantities of organic solvents, to focus on compounds of higher biological interest such as the acidic triterpenes.^9–11^ Nevertheless, at the same time, researchers working in the polymers sector have attempted the synthesis of this complex structure from its monomeric unit (myrcene), which can be abundantly sourced from diverse plant species.^12^ Indeed, poly-β-myrcene can be synthesized through various polymerization techniques, such as anionic, free radical, or coordination polymerization. Anionic polymerization, in particular, is often preferred for producing poly-β-myrcene due to its ability to control the resulting polymer’s molar mass and structural configuration. The process involves using strong bases as initiators, which cause the monomer units to link together, forming long polymer chains.^13,14^

Moreover, despite the apparent interest in this versatile material, an issue that has yet to be resolved is the accurate quantification of poly-β-myrcene inside its primary natural source, the resin of *Pistacia lentiscus*. In terms of quantification, nuclear magnetic resonance (NMR) spectroscopy is a powerful tool that may be employed since it is a nondestructive technique, where the sample does not come in contact with instrumentation, thus negating any potential problems caused by its challenging nature.^15^ By comparing the intensities of the functional group peaks with pure *cis*-1,4-poly-β-myrcene, it might be possible to estimate its concentration in CMG. Developing standardized methods for isolating and quantifying *cis*-1,4-poly-β-myrcene from mastic gum is crucial for better understanding its composition and exploring its applications in sustainable materials. As a natural polymer, *cis*-1,4-poly-β-myrcene may be an ideal candidate for biomedical use, particularly given its biocompatibility and eco-friendly properties. In fact, early evidence suggests that CMG’s polymer might play an important role in the absorption process of the resin’s pharmacologically active compounds,^16^ while recently, the potential applications of its epoxidation products were investigated.^17,18^ Consequently, further research into advanced analytical techniques is needed to unlock the full potential of this natural polymer and ensure its effective use in various industries.

Taking the above into consideration, in the current work as a first step, the production of a *cis*-1,4-poly-β-myrcene sample that may be used as a standard in the developed quantification procedure was carried out by centrifugal partition chromatography (CPC). CPC is a solid support-free liquid–liquid chromatography technique that allows the separation of compounds based on their partitioning between at least two immiscible liquid phases.^19–21^ CPC offers advantages in selectivity, sample loading capacity, and scalability, and it avoids irreversible adsorption on solid supports, making it particularly valuable for purifying natural products.^20,22,23^ The process used in this study was initially developed for fractionating neutral and acid terpenes from CMG, but it can also be used to recover the highly purified polymeric fraction. In this study, following the complete characterization of the molar mass distribution of the isolated *cis*-1,4-poly-β-myrcene by GPC, 2D ^1^H DOSY and ^1^H–^13^C edited HSQC NMR experiments were, respectively, used for its qualitative and quantitative determination in mastic samples for the first time.

## Results and Discussion

### Purification of a Standard Poly-β-myrcene Sample

As mentioned above, most researchers working on CMG have used methods based on precipitation of its natural polymer in solvents or solvent mixtures such as EtOAc/MeOH.^7–9^ However, this approach does not achieve sufficient purity for the purified *cis*-1,4-poly-β-myrcene to be used as a standard for quantification in CMG. This is why a process based on CPC has been used, based on our previous works^24,25^ aiming at isolating CMG’s neutral and acidic terpenes but also allowing recovery of the polymer fraction at the end of the process. An elution–extrusion step^26–28^ (see the Experimental Section) by pumping *n*-hexane (*n*-Hex) in the descending mode was thus carried out at the end of the process, allowing recovery of the highly apolar *cis*-1,4-poly-β-myrcene remaining in the *n*-Hex-rich organic stationary phase. This technique was made possible by the liquid nature of the stationary phase. After solvent evaporation, 385.9 mg of *cis*-1,4-poly-β-myrcene was obtained from the combined extrusion phase fraction (F16) (see Figure S1). Chemical structure and purity were confirmed by subsequent NMR experiments, confirming that CPC constitutes an excellent solution for the fast and efficient recovery of mastic’s natural polymer in a limited time frame.

### Determination of Poly-β-myrcene’s Molar Mass Using GPC

SEC and GPC have been employed only two times in the past to characterize the molar mass distribution of the polymer isolated from mastic gum.^7,8^ Nevertheless, the polymer in both studies was either isolated through precipitation or synthesized. As this study constitutes the first isolation of high-purity *cis*-1,4-poly-β-myrcene using the approach of CPC, GPC was employed for comparison purposes with the existing literature. GPC elution profiles (Experimental Section) and corresponding molar mass calculations of the isolated *cis*-1,4-poly-β-myrcene are shown in Figure 1 and Table 1. The molar mass distributions show that the RI and UV measurements cover different molar mass ranges and that UV active sample constituents generally have lower molar mass than the corresponding sample constituents detected during RI measurements.

**Figure 1:**
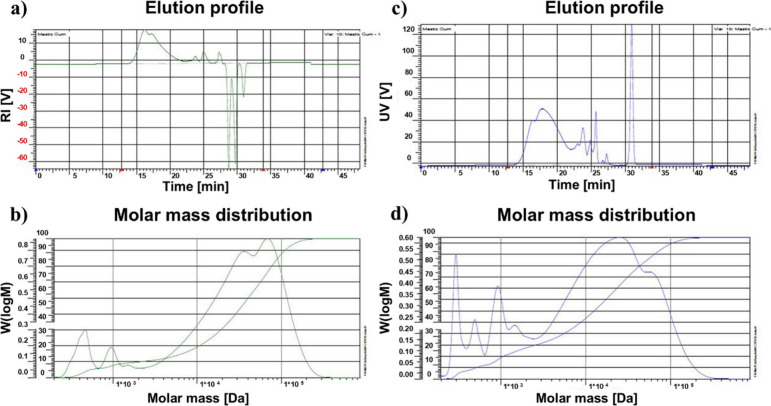
GPC analysis of poly-β-myrcene: (a) the elution profile of the sample registered by the RI detector and (b) the corresponding molar mass distributions; (c) the UV detector response of the poly-β-myrcene elution profile and (d) the corresponding molar mass distribution.

**Table 1:** Summary of the Average Molar Mass Distributions of Poly-β-myrcene, Where *M*_n_ Is the Number Average Molar Mass, *M*_w_ Is the Mass Average Molar Mass, *M*_p_ Is the Molar Mass of the Highest Peak, *M*_z_ Is the Size Average Molar Mass, All Data Are Given in Dalton, and *M*_w_/*M*_n_ Is the Polydispersity Index

Sample	Detection Method	*M*_n_ (Da)	*M*_w_ (Da)	*M*_p_ (Da)	*M*_*z*_ (Da)	Polydispersity
poly-β-myrcene	RI	4,055	45,210	68,723	86,903	11,150
	UV	2,224	26,618	26,855	73,492	11,966

The molar mass distribution pattern indicates that, in addition to the observed lower molar mass (*M*_w_) values (e.g., *M*_w_ 26,618 (Da)/46,210 (Da)), these parts of the poly-β-myrcene have more UV-active structures than the higher-molecular parts.

The GPC elution profiles with RI detection of poly-β-myrcene suggest that it contains at least seven discrete components with a rather broad molar mass distribution (polydispersity index 11,150) ranging from 1 × 10^2^ to 1 × 10^5^ Da. The mass average molar mass (*M*_w_) is calculated at 45,210 Da, indicating a predominant detection of high molecular components in line with the molar mass distribution profile. In synergy, the UV elution profile suggests the presence of at least six discrete components having molar masses between 1 × 10^1^ and 1 × 10^5^ Da, consistent with the RI data. However, the calculated mass average molar mass (*M*_w_) is determined at 26,618 Da, which is somewhat smaller than the corresponding value for RI detection, indicating that the smaller molar mass fractions of poly-β-myrcene have more UV active groups.

The mass average molar mass determined for *cis*-1,4-poly-β-myrcene is in line with reports on other solid-state terpene and nonterpene (i.e., sugar-based guar gum) based plant resins and gums, which range from 50,000 to 300,000 Da.^29^ Compared to the results of the two previous studies on *P. lentiscus*, the molar mass distribution seems to be in accordance with the one obtained from the team of van den Berg et al.^7^ but varies significantly from the study of Sharifi et al.^8^ A possible explanation could be that, while the samples of both studies are commercial, the one used by van den Berg et al. is most probably from the island of Chios, according to the manufacturer, while the latter (Sharifi et al.) is *P. lentiscus* of unknown origin.

### Analysis of Mastic Samples Focusing on Polymer Using 1D and 2D NMR Experiments

#### Qualitative ^1^H DOSY NMR Experiments

NMR spectroscopy has long been the technique of choice for the structural elucidation of natural compounds. The task is more challenging in the case of complex mixtures. So far, only a few solutions have been proposed to tackle this challenge: liquid chromatography (LC)-NMR hyphenation,^30^ multiple-quantum NMR spectroscopy,^31^ combined or not with broadband homonuclear decoupling^32^ and sparse sampling,^33^ *ViscY* NMR experiments (*Visc*osit*y* enhancement spectroscop*Y*),^34^ and diffusion-ordered spectroscopy (DOSY).^35,36^ In this work, we applied the DOSY approach to qualitatively identify the presence of the natural polymer *cis*-1,4-poly-β-myrcene in CMG samples.

The NMR DOSY experiment is a powerful method for separating and analyzing the components of a mixture based on their translational diffusion coefficients.^35,36^ In a DOSY experiment, a series of NMR spectra are recorded with varying strengths of pulsed field gradients. These gradients cause the NMR signal attenuation of molecules at rates that are dependent on their diffusion coefficients. Smaller molecules will diffuse faster than larger ones, revealing more attenuated NMR signals. These NMR signals are collected and analyzed to determine how quickly (or not) each molecule diffuses. The resulting data are processed to create a 2D DOSY spectrum. In this spectrum, one axis represents the chemical shift (δ) (as in a usual NMR spectrum), and the other axis represents the translational diffusion coefficient (*D*). This allows for the separation of signals from different molecules based on their diffusion rates.

In this context, poly-β-myrcene and CMG samples dissolved in CDCl_3_ were analyzed by ^1^H/^1^D and 2D NMR experiments. In the 2D ^1^H DOSY spectra of CMG samples, we observed that *cis*-1,4-poly-β-myrcene signals were distinguished from those of the rest of the sample. Indeed, the *cis*-1,4-poly-β-myrcene translational diffusion values (*D* ∼ 304 μm^2^/s) are much lower than those of main components such as triterpenic acids (*D* ∼ 609 μm^2^/s) and triterpenic aldehydes (*D* ∼ 841 μm^2^/s) (see Figure 2). We compared the *cis*-1,4-poly-β-myrcene 1D spectrum with the sum of the 1D slices extracted from the DOSY spectrum of a CMG sample corresponding to the polymer component. The overlay of both spectra revealed similar spectra, demonstrating the capability of the DOSY experiment to efficiently identify and separate polymer signals from the rest of the CMG signals.

**Figure 2:**
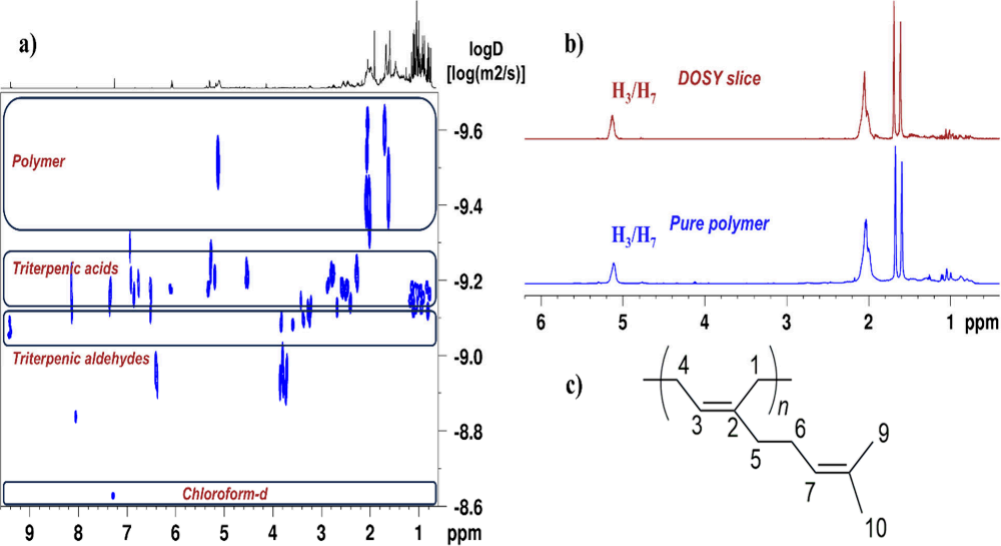
(a) 2D ^1^H DOSY spectrum of a CMG sample and (b) overlay of the sum of 1D slices (in red) extracted from the poly-β-myrcene region of the DOSY spectrum and 1D ^1^H NMR spectrum (in blue) of a pure polymer sample. (c) Chemical structure of the poly-β-myrcene.

#### Quantitative ^1^H–^13^C HSQC NMR Experiments

Quantitative NMR (qNMR) is a well-recognized method for ascertaining the concentration of one or more chemical species in solution by measuring the area beneath the NMR signal peaks due to the correlation between NMR signal areas and the number of nuclei contributing to those signals.^37–41^ This indicates that measuring the area (integral) of a peak enables the measurement of the concentration of the corresponding compound in the sample through the use of an internal or external calibrant, provided that all NMR operating and processing conditions are met (stable magnetic field, proper shimming, adequately long recycling delay, calibrated RF pulses, suitable signal-to-noise ratio, etc.). However, very congested 1D NMR spectra hinder the application of the 1D qNMR experiments. Our study revealed a substantial overlap in the 1D ^1^H spectra of CMG samples (Figure 3). A remedy for that was to consider quantitative 2D ^1^H–^13^C NMR experiments to spread the NMR information alongside a second dimension (^13^C). However, due to several experimental biases, qNMR through 2D NMR experiments is very challenging.^40^ 2D NMR cross-peak volumes are strongly molecule-dependent and site-dependent because of different *T*_1_ and *T*_2_ relaxation times and *J* coupling constants (homo- and heteronuclear). Besides, pulse sequence delays, pulse angle effects, and off-resonance effects will also affect 2D NMR signals. We can express the dependence of 2D cross-peak volumes according to *V* = *k*(*T*_1_, *T*_2_, ^*n*^ *J*_CH_, ^*n*^ *J*_HH_, delays, pulse angles, etc.) × *N* × [*C*] × *V*_s_ with *N* being the number of spins (known), *V*_s_ the sensitive coil volume (the same for all samples to analyze), [*C*] the concentration of the analyte of interest, and *k* a proportional constant depending on *T*_1_, *T*_2_, ^*n*^ *J*_CH_, ^*n*^ *J*_HH_, delays, pulse angles, etc. Finally, the long duration of 2D NMR experiments may prevent general use for high-throughput quantitative applications and affect their quantitative performance. Three approaches exist to reach the goal of quantitative NMR: (i) to modify the NMR pulse sequence to remove the dependence of *k* on 2D cross-peak volumes, (ii) to determine for each peak the value of *k* based on theoretical considerations, and (iii) to determine the value of *k* by relying on more usual analytical approaches: calibration or standard additions.

**Figure 3:**
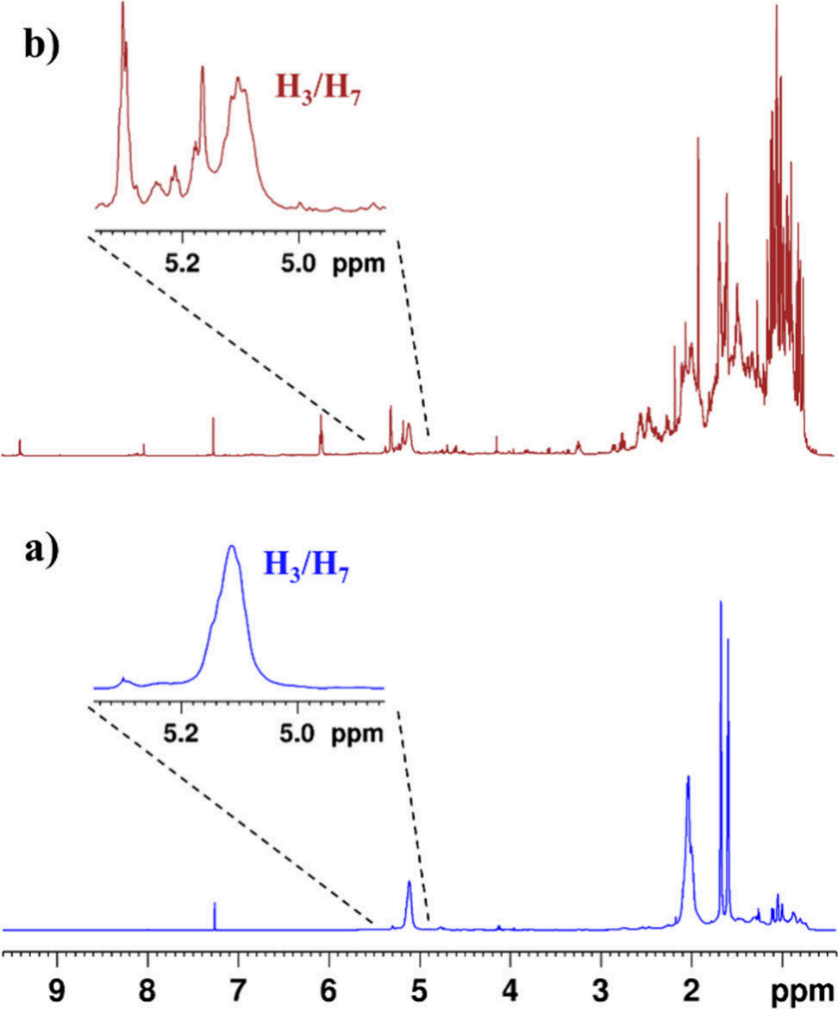
Overlay of 1D ^1^H spectra of (a) the poly-β-myrcene polymer sample (in blue) and (b) the CMG sample R10 (in red) and zoom of the ethylenic H_3_/H_7_ regions.

In this context, we considered the first approach through a dedicated heteronuclear ^1^H–^13^C HSQC experiment involving matched sweep adiabatic pulses to ensure quantitative signals and pure *cis*-1,4-poly-β-myrcene polymer as an external calibrant. We integrated the cross-peaks of ethylenic protons (H_3_/H_7_) from ^1^H–^13^C HSQC spectra of the pure polymer and 12 CMG samples collected from different areas of the island of Chios (*P. lentiscus* var. *Chia*) and one mastic sample from Iran (*P. atlantica*) (Figures 4, S2, and S3). Then, we calculated the percentage of poly-β-myrcene (*P*_CMG_) in every CMG sample (Tables 2, 3, and S2) according to the following eq 1:

1 $$P_{CMG}=\frac{I_{CMG}}{I_{cal}}\times \frac{N_{cal}}{N_{CMG}}\times \frac{M_{CMG}}{M_{cal}}\times \frac{m_{cal}}{m_{CMG}}\times P_{cal}$$

*I* = integral, *N* = number of spins belonging to the respective molecular unit, *M* = molar mass in g mol^–1^, *m* = mass in g, and *P*(cal) = purity of the poly-β-myrcene polymer in % g/g.

**Figure 4:**
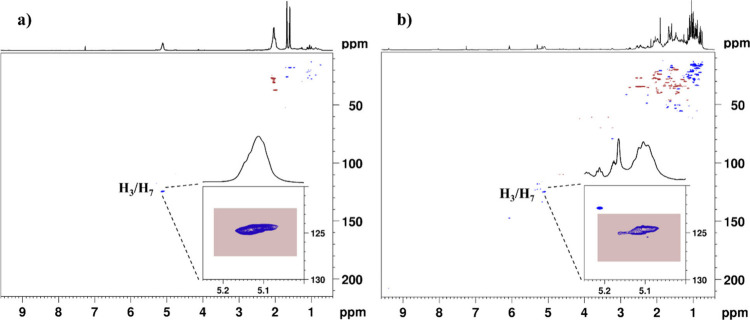
^1^H–^13^C HSQC spectra (using the *hsqcedetgpsp.3* pulse sequence from the Bruker library) of (a) the poly-β-myrcene polymer and (b) the CMG sample R10 and zoom of ethylenic H_3_/H_7_ areas integrated for calculation.

**Table 2:** Example of Poly-β-myrcene Polymer Content Calculation in CMG Sample R10 through 2D ^1^H–^13^C HSQC Experiments

External Calibrant – Pure Polymer			CMG Sample R10		
Molar Mass (g mol^–1^)	164.292		Molar Mass (g mol^–1^)	164.292	
Chemical Shift of Ethylenic H_3_/H_7_	ν(F1) [ppm]	125.01	Chemical Shift of Ethylenic H_3_/H_7_	ν(F1) [ppm]	125.01
	ν(F2) [ppm]	5.11		ν(F2) [ppm]	5.11
Mass (g)	0.01434		Mass (g)	0.01508	
Volume (L)	0.00150		Volume (L)	0.00065	
Integral [rel]	2		Integral [rel]	2	
Integral [abs]	791370000		Integral [abs]	368170000	
% Purity (g/g)	100.00		% Polymer Content (g/g)	17.62	

**Table 3:** Poly-β-myrcene Polymer Content (%) in 13 Mastic Samples Determined through 2D ^1^H–^13^C HSQC Experiments

Mastic Sample Code	Polymer Content (%, g/g)
R01	24.49
R02	15.29
R03	29.51
R04	26.35
R05	26.54
R06	32.50
R07	33.09
R08	23.28
R09	23.24
R10	17.62
R11	27.36
R12	29.16
R13	4.97

The content of the *cis*-1,4-poly-β-myrcene polymer in the 12 CMG samples (R01–R12) collected from the island of Chios covered a range from 15.29% to 33.09% (Table S2). Studies today have produced an estimate of this value at around 25–30%.^3,9,42^ However, none of these methods were quantitative; rather, they were based on extraction yield after isolating the polymer from CMG. Therefore, this study comprises the first analytical effort for quantitatively determining polymer content in CMG.

A similar resin can also be obtained from other *Pistacia* sp., the most common being *Pistacia atlantica*. This resin is often used as an adulterant in products under the label of CMG.^3^ To take it a step further, we obtained and analyzed a mastic sample originating from Iran, obtained from a different species (*P. atlantica*; R13), aiming to detect any potential variation in polymer content compared to that of *P. lentiscus* var. *Chia* (R01–R12). Indeed, sample R13 was found to contain only 4.97% polymer, significantly lower than that of all CMG samples. According to the one study that exists to this day concerning the polymer content in *P. atlantica*, Sharifi and co-workers have determined a value between 13.8% and 20.0%, depending on the subspecies, and significantly lower than a sample belonging to *P. lentiscus* (35.2%).^8^ Nevertheless, it should be noted that all calculations were once again made based on the extraction yield of the fraction obtained through decantation and not through a quantitative analytical method.

To summarize, through quantitative 2D ^1^H–^13^C HSQC NMR experiments, we established the capability to determine the poly-β-myrcene polymer content in CMG samples quickly. Analysis of additional samples from different *Pistacia* sp. in the future could also potentially highlight the polymer content as a key factor for discriminating CMG, a premium-quality PDO product, from other inferior-quality resins.^7^

## Conclusion

In summary, *cis*-1,4-poly-β-myrcene was successfully isolated for the first time using CPC, an alternative method to commonly employed decantation. This technique yielded a highly pure polymer, as confirmed by subsequent NMR analyses, demonstrating that CPC offers a rapid and efficient approach for recovering mastic’s natural polymer within a limited time frame. The purified polymer was further characterized via GPC to determine its molar mass distribution, which aligned with the only study to date that, to the best of our knowledge, utilized *cis*-1,4-poly-β-myrcene isolated from mastic gum from the island of Chios.

Beyond structure and purity determination, NMR spectroscopy was employed both qualitatively and quantitatively to further investigate the polymer within the CMG. Using 2D ^1^H DOSY NMR experiments, we effectively identified and distinguished polymer signals from the rest of the CMG matrix. Additionally, quantitative 2D ^1^H–^13^C HSQC NMR experiments enabled the rapid determination of the *cis*-1,4-poly-β-myrcene content in CMG samples. Future analysis of samples from different *Pistacia* species could further establish the polymer content as a crucial factor for differentiating CMG from lower-grade resins. This is the first time that the combination of DOSY and HSQC analysis is employed, not only for mastic but for natural resins in general. The complete proposed workflow could also be potentially applied to other food matrices.

## Experimental Section

### Materials and Chemicals

The Chios Mastiha Growers’ Association kindly provided CMG crude resin. For the isolation of poly-β-myrcene, chromatography grade water, EtOH, *n*-Hex, and EtOAc were purchased from Carlo Erba Reagents (Val de Reuil, France). TLC plates (silica gel 60 F_254_) and the reagents vanillin, sulfuric acid, triethylamine (Et_3_N), trifluoroacetic acid (TFA), and MeOH of reagent grade were purchased from Merck (Rahway, NJ, USA). Deuterated chloroform used for NMR analysis was acquired from Merck SA (Athens, Greece), while NMR tubes (D600-5-7, 5 mm diameter, and 7 in. length) with polytetrafluoroethylene (PTFE) caps were obtained by Deutero GmbH (Kastellaun, Germany).

### General Experimental Procedures

#### Poly-β-myrcene Purification by Centrifugal Partition Chromatography

Poly-β-myrcene was purified by CPC using a lab-scale FCPE300 apparatus (Rousselet Robatel Kromaton, Annonay, France). The total column volume was 303.5 mL and composed of 7 partition disks. Each disk contains 33 twin cells (∼1 mL per twin cell) arranged circumferentially and connected by ducts with a width of 0.8 mm. The stationary phase was maintained inside the column by application of a constant centrifugal force field generated by the rotor around a single central axis. The rotation speed can be adjusted from 200 to 2000 rpm, producing a centrifugal force field in the partition twin-cell up to 437*g*. The liquid phases were pumped by a Lab Alliance Flash 100 preparative pump (State College, PA, USA). Fractions were collected by a Buchi B-684 semiautomated collector (Flawil, Switzerland).

The CPC separation process consists of three consecutive sequences that were implemented during the same run. This process was developed to isolate the main class of metabolites in CMG (i.e., the acidic triterpenes, the neutral triterpenes, and the polymeric fraction).^24,25^ The first part of the process uses the pH-zone refining mode^43–45^ to recover acidic triterpenes. The first biphasic solvent system (S1) consisted of *n*-Hex/EtOAc/EtOH/H_2_O, 8:2:5:5 (*v/v/v/v*). The upper organic phase was acidified with 100 mM TFA as a retainer, while the aqueous phase was alkalized with 80 mM Et_3_N as a displacer. The column was filled with the organic stationary phase at a flow rate of 20 mL/min and a rotation speed of 200 rpm. The sample (3 g of CMG) was dissolved in 10 mL of the aqueous alkalized phase and 10 mL of the acid-free organic phase. The rotation speed was then increased to 900 rpm, and the sample solution was injected at a rate of 20 mL/min through a Rheodyne valve equipped with a 20 mL sample loop. Subsequently, the alkalized lower phase (mobile phase) was pumped at a flow rate of 20 mL/min, and 15 fractions of 30 mL were collected. A three-step gradient elution section then followed the pH-zone refining section. For this purpose, the acid-free aqueous lower phases of systems S2 (*n*-Hex/EtOAc/EtOH/H_2_O, 8:2:7:3, *v/v/v/v*), S3 (*n*-Hex/EtOAc/EtOH/H_2_O, 8:2:8:2 *v/v/v/v*), and S4 (*n*-Hex/EtOAc/EtOH/H_2_O, 8:2:9:1, *v/v/v/v*) were pumped successively during 15 min (10 fractions) for each different mobile phase. Finally, an extrusion step was performed by pumping *n*-Hex in the descending mode,^28^ providing 10 additional fractions. Fractions were then spotted on TLC plates, sprayed with sulfuric vanillin derivatization reagent (i.e., 5% *w/v* vanillin in MeOH/5% *v/v* H_2_SO_4_ in MeOH 1:1 *v/v*) and grouped according to their TLC profile, to yield a total of 16 final fractions (F1–F16) (Figure S1). F16, corresponding to the extrusion section, which lasted 15 min, contains 385.9 mg of poly-β-myrcene whose purity is confirmed by NMR analysis after solvent evaporation. This sample will be used as a standard to develop the quantification method directly on the ground CMG.

#### GPC Analysis

The poly-β-myrcene polymer was subjected to GPC to assess its molar mass distribution. The sample was dissolved in THF for 24 h and filtered (0.45 μm) to remove small particles. GPC chromatography was carried out using an Agilent Infinity 1260 Series chromatography system equipped with an Agilent Infinity 1260 refractive index detector and an Agilent Infinity 1260 II UV detector. The clear sample was injected (*V*_i_ = 100 μL) at a concentration of 1 g/L and eluted with 100% THF at a flow rate of 1 mL/min (*T* = 40 °C). The molar mass distribution was calibrated using polystyrol molar mass markers (Agilent, Germany).^46^ The polymer sample was eluted over a series of Agilent PL gel matrix columns (PLgel Mixed D (35-38), PLgel Mixed D (39-13), PLgel Mixed E (−), Agilent, Germany). Chromatograms were detected using refractive index and UV adsorption at 254 nm.

#### NMR Spectroscopy

All NMR experiments were recorded at 298 K on a Bruker AVANCEIII 600 NMR spectrometer (Bruker BioSpin AG, Fällanden, Switzerland) operating at a proton frequency of 600.13 MHz (*B*_0_ = 14.1 T) and equipped with a *z*-gradient inverse detection 5 mm probe using the TOPSPIN software. Gradient pulses (maximum 0.535 T/m) were generated by a 10 A amplifier. Temperature was controlled using a Bruker variable temperature (BVT) unit supplied with chilled air produced by a Bruker cooling unit (BCU). All spectra of pure polymers and CMG samples were referenced so that the residual proton and carbon signal of CDCl_3_ were respectively observed at 7.26 ppm (*δ* ^*1*^ *H*) and 77.16 ppm (*δ* ^*13*^ *C*). Additional NMR data acquisition and processing parameters for Figures 2–4 are reported in the Supporting Information.

## Supplementary Material



## Notes

TLC chromatogram of the fractions after CPE separation; table of all mastic samples used for NMR analyses; additional NMR data acquisition and processing parameters for Figures 2–4; ^1^H–^13^C HSQC spectra of all mastic samples; calculations of poly-β-myrcene polymer content (%) in 13 mastic samples using quantitative ^1^H–^13^C HSQC experiments and the pure poly-β-myrcene polymer as an external calibrant (PDF)
