# Supplementary material for: Unlocking the Potential of Water-Insoluble Natural Polymers: Isolation, Characterization, and 2D NMR Quantification of cis-1,4-Poly-β-myrcene in Chios Mastic Gum
Source: J Nat Prod. 2025 Apr 14;88(8):1879–86. doi: 10.1021/acs.jnatprod.5c00256 (PMC12379139; doi:10.1021/acs.jnatprod.5c00256)
Supplement: Supplementary file 1 [file np5c00256_si_001.pdf]

SUPPORTING INFORMATION for

Unlocking the potential of water-insoluble natural polymers: Isolation, characterization, and 2D NMR quantification of *cis*-1,4-poly-β-myrcene in Chios mastic gum

Stavros Beteinakis[1]‡, Eleni V. Mikropoulou[1]‡, Dimitris Michailidis[2], Apostolis Angelis[1], Martina Haack[3], Marion Ringel[3], Thomas Brück[3], Dieter W. Brück[3], Jean-Hugues Renault[4], Alexios-Leandros Skaltsounis[1], Pedro Lameiras[4], Maria Halabalaki[1*]

[1]*Division of Pharmacognosy and Natural Products Chemistry, Department of Pharmacy, National and Kapodistrian University of Athens, Panepistimioupoli Zografou, 15771 Athens, Greece.*

[2]*PharmaGnose S.A., 57th km Athens-Lamia National Road, Oinofyta, 32011, Greece*

[3]*Werner Siemens—Chair of Synthetic Biotechnology, Department of Chemistry, School of Natural Sciences, Technical University of Munich, 85748 Garching, Germany.*

[4] *University of Reims Champagne-Ardenne, ICMR 7312, 51687 Reims, France*

‡These authors contributed equally.

***Corresponding Author:** Maria Halabalaki – E-mail: mariahal@pharm.uoa.gr, Phone: +302107274781; +306987832325

Table of contents

Subject	page S2
TLC chromatogram of the fractions after CPE separation	**page S3**
Table of all mastic samples used for NMR analyses	**page S3**
Additional NMR data acquisition and processing parameters for Figures 2 → 4	**page S3**
^{1}H-^{13}C HSQC spectra of all mastic samples	**page S5**
Calculations of poly-*β*-myrcene polymer content (%) in 13 mastic samples using quantitative ^{1}H-^{13}C HSQC experiments and the pure poly-*β*-myrcene polymer as an external calibrant	**page S7**

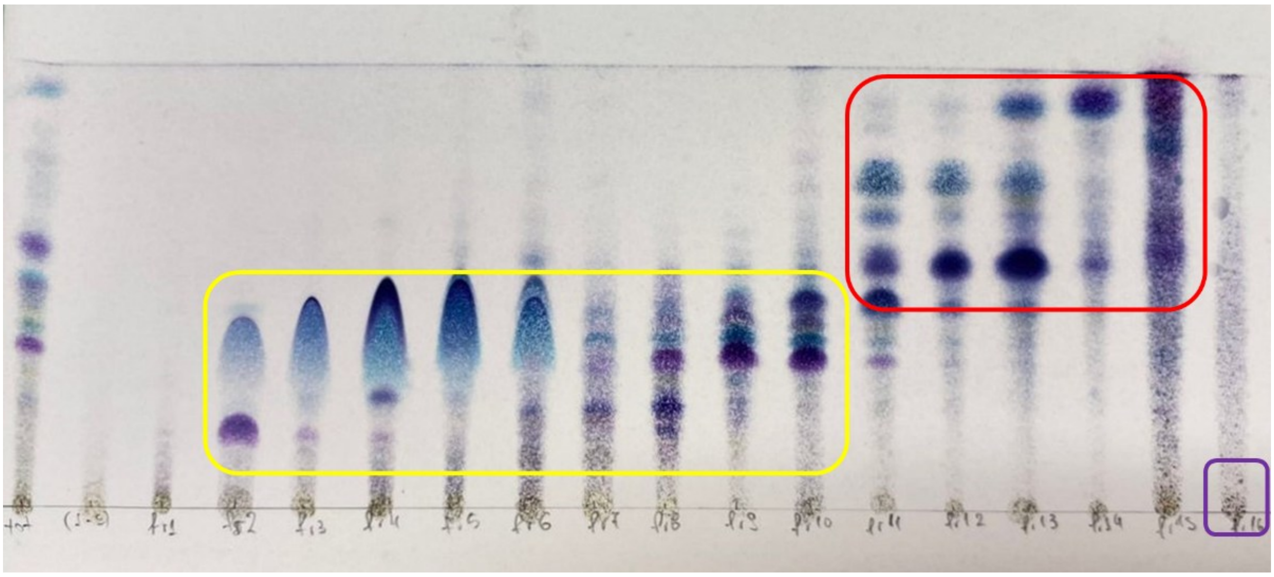

Figure S1. TLC plate showing the separation of CMG compounds after CPE pH zone refining followed by step gradient elution mode (yellow: acidic terpenes, red: neutral terpenes, purple: polymer).

Table S1. Sample code, botanical and geographical origin of all samples used for NMR analysis.

Mastic Sample Code	Botanical Origin	Geographical Origin
R01	*Pistacia lentiscus* var. *Chia*	Chios island
R02	*Pistacia lentiscus* var. *Chia*	Chios island
R03	*Pistacia lentiscus* var. *Chia*	Chios island
R04	*Pistacia lentiscus* var. *Chia*	Chios island
R05	*Pistacia lentiscus* var. *Chia*	Chios island
R06	*Pistacia lentiscus* var. *Chia*	Chios island
R07	*Pistacia lentiscus* var. *Chia*	Chios island
R08	*Pistacia lentiscus* var. *Chia*	Chios island
R09	*Pistacia lentiscus* var. *Chia*	Chios island
R10	*Pistacia lentiscus* var. *Chia*	Chios island
R11	*Pistacia lentiscus* var. *Chia*	Chios island
R12	*Pistacia lentiscus* var. *Chia*	Chios island
R13	*Pistacia atlantica*	Iran

Additional NMR data acquisition and processing parameters for Figures 1 -> 3:

Figure 1. a) 2D ^{1}H DOSY spectrum of a CMG sample dissolved in pure $CDCl_3$ and b) overlay of the sum of 1D slices (in red) extracted from the poly-β-myrcene region of the DOSY spectrum and 1D NMR spectrum (in blue) of the poly-β-myrcene polymer sample, c) chemical structure of the poly-β-myrcene. Data were acquired by means of the ledbpgp2s pulse sequence, at 298 K, at 600 MHz (^{1}H). The diffusion time (Δ) was 50 ms and the gradient pulse length (δ) was 1.6 ms. The size of the raw data set was 32 x 32,768 with 8 scans per FID, and a 12 s relaxation delay (D_1), resulting in a 61.10 min recording time. The gradient intensity values were equally spaced from 2% to

98%. The DOSY spectrum was calculated using the Bruker TOPSPIN Software. The monoexponential curve fitting tool was used to calculate the indirectly detected dimension using Dynamics Center (Bruker BioSpin AG, Fällanden, Switzerland). Log (*D*) was calculated with *D* expressed in $(\mu m)^2/s$.

Figure 2. Overlay of 1D ^1H spectra of a) the poly-*β*-myrcene polymer sample (in blue), b) the CMG sample R10 (in red), both dissolved in pure $CDCl_3$ and zoom of ethylenic H_3/H_7 regions. 1D ^1H spectra were recorded with NS = 16 scans, a 30 s recycling delay (D_1), at 298 K, at 600 MHz (^1H). The FIDs (128k points, spectral width = 9014.42 Hz) were zero-filled to 128k points.

Figure 3. a) 2D ^1H-^{13}C HSQC spectra (using the *hsqcedetgpsp.3* pulse sequence from the Bruker library) of a) the poly-*β*-myrcene polymer, b) the CMG sample RS10 (both dissolved in pure $CDCl_3$) and zoom of ethylenic H_3/H_7 areas integrated for calculation, at 298 K, at 600 MHz (^1H). Data matrix was recorded in Echo-Antiecho mode with Non-Uniform Sampling equal to 50%; its size was 1k × 4k with 8 scans per FID, a 1.5 s relaxation delay, resulting in a 124.15 min recording time. The spectral widths were respectively 7211.54 Hz in ^1H dimension and 36219.45 Hz in ^{13}C dimension. Data matrix was multiplied in both dimensions by a shifted squared sine bell function (SSB = 2) before zero filling to a 1k × 4k size. Processing mode using Multidimensional Decomposition (MDD) with the amount of sparse sampling equal to 50%.

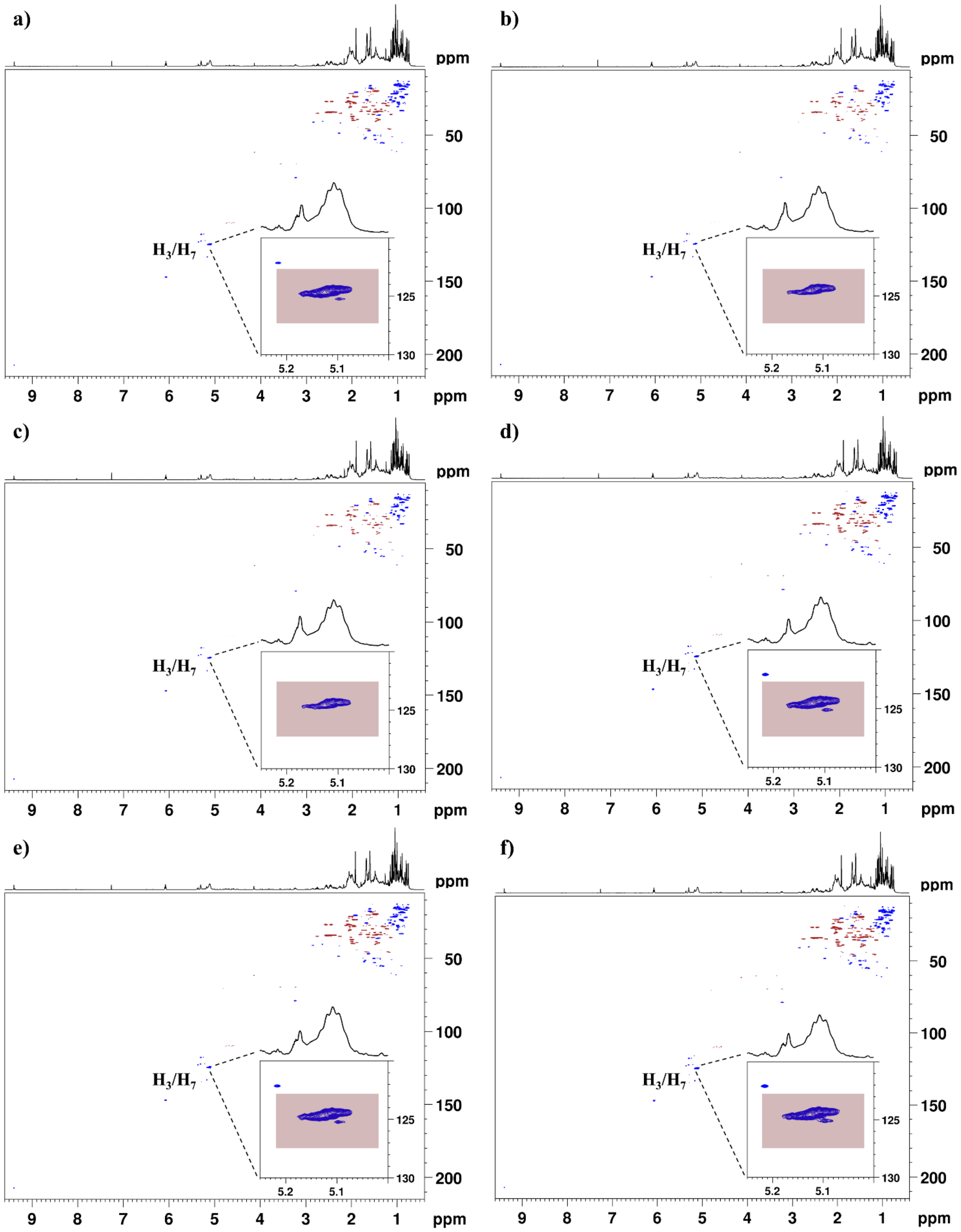

Figure S2. 2D ^{1}H-^{13}C HSQC spectra with zoom of ethylenic H_3/H_7 areas integrated for calculation. (using the *hsqcedetgpsp.3* pulse sequence from the Bruker library) of a) CMG sample R01, b) CMG sample R02, c) CMG sample R03, d) CMG sample R04, e) CMG sample R05, f) CMG sample R06.

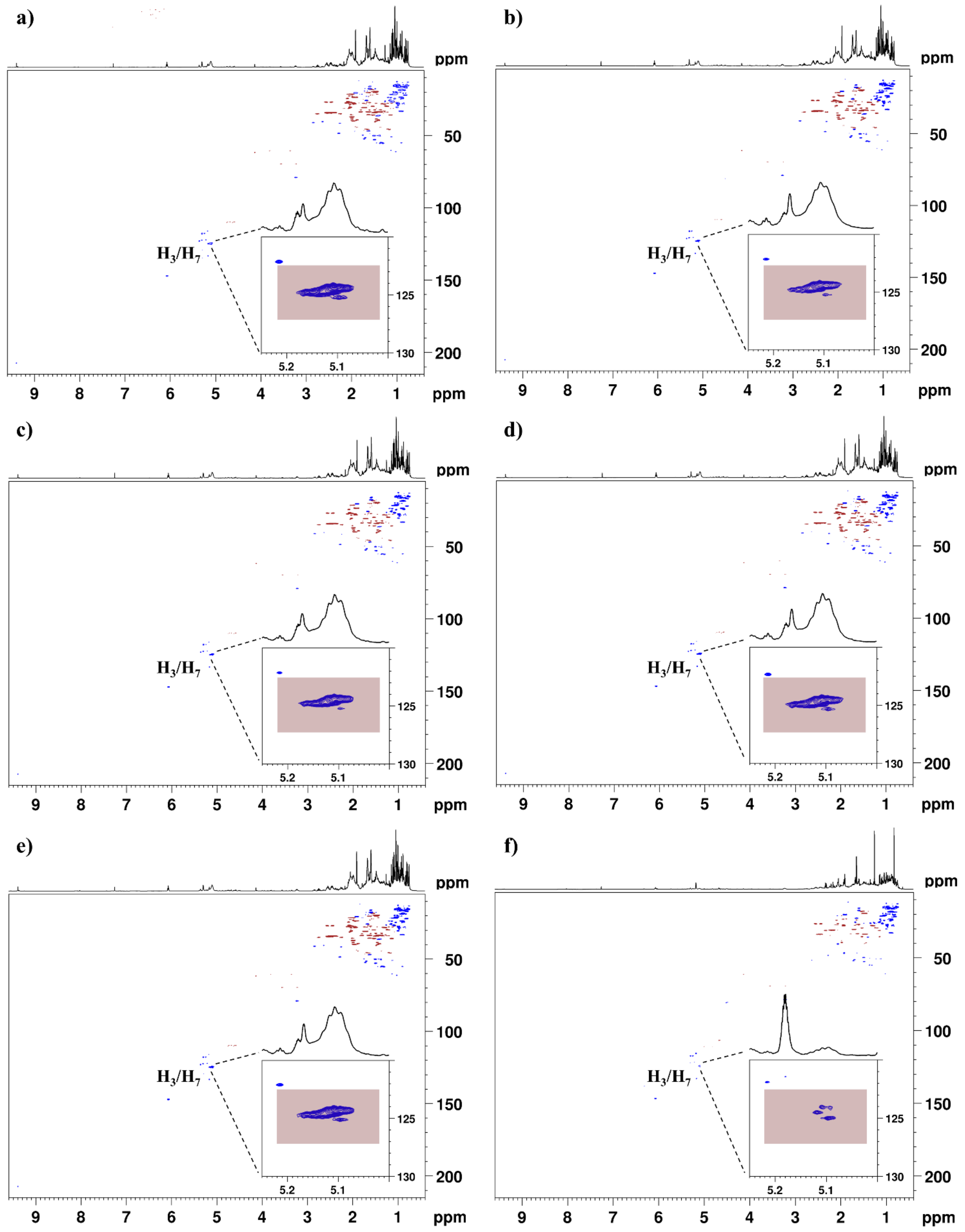

Figure S3. 2D ^{1}H-^{13}C HSQC spectra with zoom of ethylenic H_3/H_7 areas integrated for calculation. (using the *hsqcedetgpsp.3* pulse sequence from the Bruker library) of a) CMG sample R07, b) CMG sample R08, c) CMG sample R09, d) CMG sample R11, e) CMG sample R12, f) mastic sample from Iran R13.

Table S2. Calculations of poly-β-myrcene polymer content (%, g/g) in 13 mastic samples using quantitative ^{1}H-^{13}C HSQC experiments and the pure poly-β-myrcene polymer as an external calibrant.

Sample Code: R01								
**External Calibrant - Pure Polymer**	Molar Mass (g mol^{-1})	164.292		Mass (g)	0.01434	Volume (L)	0.00150	
	Integral [abs]	Integral [rel]	ν(F1) [ppm]	ν(F2) [ppm]				
	791370000	2	125.01	5.11		% Purity (g/g)		100.00
**Mastic Sample**	Molar Mass (g mol^{-1})	164.292		Mass (g)	0.01564	Volume (L)	0.00065	
	Integral [abs]	Integral [rel]	ν(F1) [ppm]	ν(F2) [ppm]				
	487720000	2	125.11	5.11		**% Polymer Content (g/g)**		**24.49**
Sample Code: R02								
**External Calibrant - Pure Polymer**	Molar Mass (g mol^{-1})	164.292		Mass (g)	0.01434	Volume (L)	0.00150	
	Integral [abs]	Integral [rel]	ν(F1) [ppm]	ν(F2) [ppm]				
	791370000	2	125.01	5.11		% Purity (g/g)		100.00
**Mastic Sample**	Molar Mass (g mol^{-1})	164.292		Mass (g)	0.01535	Volume (L)	0.00065	
	Integral [abs]	Integral [rel]	ν(F1) [ppm]	ν(F2) [ppm]				
	298890000	2	125.05	5.11		**% Polymer Content (g/g)**		**15.29**
Sample Code: R03								
**External Calibrant - Pure Polymer**	Molar Mass (g mol^{-1})	164.292		Mass (g)	0.01434	Volume (L)	0.00150	
	Integral [abs]	Integral [rel]	ν(F1) [ppm]	ν(F2) [ppm]				
	791370000	2	125.01	5.11		% Purity (g/g)		100.00
**Mastic Sample**	Molar Mass (g mol^{-1})	164.292		Mass (g)	0.01444	Volume (L)	0.00065	
	Integral [abs]	Integral [rel]	ν(F1) [ppm]	ν(F2) [ppm]				
	542600000	2	125.11	5.11		**% Polymer Content (g/g)**		**29.51**
Sample Code: R04								
**External Calibrant - Pure Polymer**	Molar Mass (g mol^{-1})	164.292		Mass (g)	0.01434	Volume (L)	0.00150	
	Integral [abs]	Integral [rel]	ν(F1) [ppm]	ν(F2) [ppm]				
	791370000	2	125.01	5.11		% Purity (g/g)		100.00
**Mastic Sample**	Molar Mass (g mol^{-1})	164.292		Mass (g)	0.01520	Volume (L)	0.00065	
	Integral [abs]	Integral [rel]	ν(F1) [ppm]	ν(F2) [ppm]				
	510080000	2	125.02	5.11		**% Polymer Content (g/g)**		**26.35**

Sample Code: R05								
**External Calibrant - Pure Polymer**	Molar Mass (g mol^{-1})	164.292		Mass (g)	0.01434	Volume (L)	0.00150	
	Integral [abs]	Integral [rel]	ν(F1) [ppm]	ν(F2) [ppm]				
	791370000	2	125.01	5.11		% Purity (g/g)		100.00
**Mastic Sample**	Molar Mass (g mol^{-1})	164.292		Mass (g)	0.01556	Volume (L)	0.00065	
	Integral [abs]	Integral [rel]	ν(F1) [ppm]	ν(F2) [ppm]				
	525950000	2	125.13	5.11		**% Polymer Content (g/g)**		**26.54**
Sample Code: R06								
	Molar Mass (g mol^{-1})	164.292		Mass (g)	0.01434	Volume (L)	0.00150	
**External Calibrant - Pure Polymer**	Integral [abs]	Integral [rel]	ν(F1) [ppm]	ν(F2) [ppm]				
	791370000	2	125.01	5.11		% Purity (g/g)		100.00
	Molar Mass (g mol^{-1})	164.292		Mass (g)	0.01528	Volume (L)	0.00065	
**Mastic Sample**	Integral [abs]	Integral [rel]	ν(F1) [ppm]	ν(F2) [ppm]				
	632400000	2	125.02	5.11		**% Polymer Content (g/g)**		**32.50**
Sample Code: R07								
	Molar Mass (g mol^{-1})	164.292		Mass (g)	0.01434	Volume (L)	0.00150	
**External Calibrant - Pure Polymer**	Integral [abs]	Integral [rel]	ν(F1) [ppm]	ν(F2) [ppm]				
	791370000	2	125.01	5.11		% Purity (g/g)		100.00
	Molar Mass (g mol^{-1})	164.292		Mass (g)	0.01527	Volume (L)	0.00065	
**Mastic Sample**	Integral [abs]	Integral [rel]	ν(F1) [ppm]	ν(F2) [ppm]				
	643570000	2	125.08	5.11		**% Polymer Content (g/g)**		**33.09**
Sample Code: R08								
	Molar Mass (g mol^{-1})	164.292		Mass (g)	0.01434	Volume (L)	0.00150	
**External Calibrant - Pure Polymer**	Integral [abs]	Integral [rel]	ν(F1) [ppm]	ν(F2) [ppm]				
	791370000	2	125.01	5.11		% Purity (g/g)		100.00
	Molar Mass (g mol^{-1})	164.292		Mass (g)	0.01498	Volume (L)	0.00065	
**Mastic Sample**	Integral [abs]	Integral [rel]	ν(F1) [ppm]	ν(F2) [ppm]				
	444140000	2	125.10	5.11		**% Polymer Content (g/g)**		**23.28**

Sample Code: R09								
**External Calibrant - Pure Polymer**	Molar Mass (g mol^{-1})	164.292		Mass (g)	0.01434	Volume (L)	0.00150	
	Integral [abs]	Integral [rel]	ν(F1) [ppm]	ν(F2) [ppm]				
	791370000	2	125.01	5.11		% Purity (g/g)		100.00
**Mastic Sample**	Molar Mass (g mol^{-1})	164.292		Mass (g)	0.01544	Volume (L)	0.00065	
	Integral [abs]	Integral [rel]	ν(F1) [ppm]	ν(F2) [ppm]				
	457020000	2	125.11	5.11		**% Polymer Content (g/g)**		**23.24**
Sample Code: R10								
**External Calibrant - Pure Polymer**	Molar Mass (g mol^{-1})	164.292		Mass (g)	0.01434	Volume (L)	0.00150	
	Integral [abs]	Integral [rel]	ν(F1) [ppm]	ν(F2) [ppm]				
	791370000	2	125.01	5.11		% Purity (g/g)		100.00
**Mastic Sample**	Molar Mass (g mol^{-1})	164.292		Mass (g)	0.01508	Volume (L)	0.00065	
	Integral [abs]	Integral [rel]	ν(F1) [ppm]	ν(F2) [ppm]				
	338380000	2	125.11	5.11		**% Polymer Content (g/g)**		**17.62**
Sample Code: R11								
**External Calibrant - Pure Polymer**	Molar Mass (g mol^{-1})	164.292		Mass (g)	0.01434	Volume (L)	0.00150	
	Integral [abs]	Integral [rel]	ν(F1) [ppm]	ν(F2) [ppm]				
	791370000	2	125.01	5.11		% Purity (g/g)		100.00
**Mastic Sample**	Molar Mass (g mol^{-1})	164.292		Mass (g)	0.01544	Volume (L)	0.00065	
	Integral [abs]	Integral [rel]	ν(F1) [ppm]	ν(F2) [ppm]				
	537950000	2	125.05	5.11		**% Polymer Content (g/g)**		**27.36**
Sample Code: R12								
**External Calibrant - Pure Polymer**	Molar Mass (g mol^{-1})	164.292		Mass (g)	0.01434	Volume (L)	0.00150	
	Integral [abs]	Integral [rel]	ν(F1) [ppm]	ν(F2) [ppm]				
	791370000	2	125.01	5.11		% Purity (g/g)		100.00
**Mastic Sample**	Molar Mass (g mol^{-1})	164.292		Mass (g)	0.01549	Volume (L)	0.00065	
	Integral [abs]	Integral [rel]	ν(F1) [ppm]	ν(F2) [ppm]				
	575210000	2	125.06	5.11		**% Polymer Content (g/g)**		**29.16**

Sample Code: R13								
**External Calibrant - Pure Polymer**	Molar Mass (g mol^{-1})	164.292		Mass (g)	0.01434	Volume (L)	0.00150	
	Integral [abs]	Integral [rel]	ν(F1) [ppm]	ν(F2) [ppm]				
	791370000	2	125.01	5.11		% Purity (g/g)		100.00
**Mastic Sample**	Molar Mass (g mol^{-1})	164.292		Mass (g)	0.01544	Volume (L)	0.00065	
	Integral [abs]	Integral [rel]	ν(F1) [ppm]	ν(F2) [ppm]				
	97812000	2	125.12	5.11		**% Polymer Content (g/g)**		**4.97**
